# The History of Tuberculosis

**Published:** 1885-07

**Authors:** Albert Johne


### ART. XXI.—THE HISTORY OF TUBERCULOSIS.

BY DR. ALBERT JOHNE.

[Continued from page 188.]

What consequence does the proof of the identity and infectiousness of Tuberculosis in man and animals have for the medical and veterinary police?

When we take into consideration the proven possibility of the transmission of tuberculosis to human beings through the consumption of flesh and milk from tuberculous animals, we see at once that Gerlach has clearly indicated the task of the veterinary police. The government must forbid the sale of flesh, even as much as they should the milk from such animals.

That such a procedure is justifiable has been still more strongly proven by the more recent researches in this direction. Fear of the consequences seems to have been the cause of the greatest reticence, as well as prudence in the consideration of these questions. From the practical stand-point no one has yet gone to the extent demanded by Gerlach in the execution of laws for the regulation of the sale and disposal of such food.

It is doubtful if the veterinary police have given the attention to this question which its public importance demands. Statistics in this direction are decidedly wanting in all parts of Germany, with the exception of Bavaria. The governments of Swabia and Neuburg, through the influence of the Veterinary Association, have made regulations requiring all official veterinary inspectors to gather statistics with reference to every case of tuberculosis, or perlzucht in cattle in their respective districts, and also such observations with reference to the injuriousness of the flesh or milk from such animals as they may come across. They have also to notify the doctors of such districts of the families where such cattle are found, especially milk cows, in order that they may assist in such observations. The position which the medical police at present assume towards the question of the use of the flesh of tuberculous cattle is as follows: Such meat must be classed among those articles which are considered as injurious to the public health as food. The exact execution of laws of this nature would have a most deleterious effect upon the owners of marketable animals. Experience has shown that cattle having tubercular processes in various organs can still be in good condition for market, and yield a first-class (*looking*) article of food at the shambles; this is especially the case when the complications are almost entirely limited to the serous membranes. When the tuberculous masses are removed from these membranes it frequently happens that the balance of the carcass presents an entirely blameless appearance. The question is:

Shall such meat be inconsiderately thrown away? Where shall the limits be drawn between what is suitable and non-suitable for human food?

To properly answer these questions is a matter requiring the greatest circumspection. Semmer concludes "that the perlzucht and tuberculosis are either uninjurious as human food, and then all can be eaten, or it is injurious, in which case the sale of such material should be absolutely forbidden," (but he leaves the government experts to decide which.)

Gerlach does not put it quite so strongly, but strong enough, however, to decidedly interfere with the agriculturists and breeders.

According to him the disease must be assumed to have become constitutional, *i. e.*, generally when the animals begin to fail in condition when dietetic causes are unknown. The animals are then to be considered unfit for the shambles, hence, unfit for human food.

1st. When the lymph glands in the vicinity of the tuberculous diseased organs have become infected, thus rendering possible the extension of the disease over the organism by way of the lymph circulation. When the lymph glands are not effected the processes of the disease still remain localized. When a number of lymph glands have become affected the entire lymphatic system may be suspected.

2. When cheesy-degeneration has begun in the lungs or elsewhere, the more cheesy-tubercles, the more dangerous the flesh is to be considered.

3. When emaciation has begun.

4. When a general tuberculosis can be diagnosticated.

It has been objected that Gerlach's requirements extend far beyond the grade of actual necessity, as in many cases finely-conditioned animals would have to be withdrawn from the market were they to be resorted to.

The question, however, in reality, is not of so unfavorable a nature as Gerlach and some others would have us believe. There is no question as to the infectiousness of tuberculosis. That it is a disease which is produced by specific virus capable of producing tubercles in any tissue with which it comes in contact. Cohnheim has shown the manner in which this virus extends over the organism through natural channels, partly along the inner surface of canals having a mucous lining, partly through the lymph glands and ducts, or from the intestines through the radicles of the portal circulation. His and other investigations have further shown that this virus can gain access to the circulation either from a primary or secondarily diseased organ, and thereby carried to all parts of the system, causing a generalization of the disease per metastosis. If this is but seldom the way, the reason, according to Cohnheim, is to be sought in the fact that the quantity of virus thus circulating is too small to be effective.

On account of the results of Koch's investigations we must conclude, also that small quantities would be much more likely to be removed from the organism by means of the excretory organs before they had time to develop their activities. On the contrary, generalization would be likely to occur in instances when large quantities of the virus are rapidly added to the blood. It has already been mentioned that the thoracic duct and pulmonary veins form the two great supply items to the circulation in this direction.

Other authorities have called attention to the fact that such general infection occurs all the more readily the more the tuberculous processes have undergone cessation, and become softened. Consequently, when the tuberculous processes have a prevailing fibroid character, or calcify quickly, or are formed in a firm stromo, the less liability there is to generalization, *i. e.*, organization of the virus.

These assertions have been fully confirmed by clinical observations. From the above it is to be seen that we can only speak of infection of the blood, when the phenomena indicate that generalization of the disease has occurred, *i. e.*, when aside from primary and secondarily diseased centres, others are present, have no direct connection with them, and which could only have become infected through the mediation of the circulation.

What are the conditions in the muscles, *i. e.*, the flesh we eat? In what way can they become infected?

The anatomy gives the answer. Only through the blood. Neither of the lymph systems of the breast, abdomen or pelvic organs, nor those of the serus membranes of these cavities have their course in the great muscle-groups, and therefore cannot convey infection to them, except to an infinitesimal degree in some small localities, as the intercostales.

The question at what period in the disease is the flesh of tuberculous animals to be looked upon as infected, and hence itself to be considered as infected also, does not depend, as Gerlach has asserted, upon the complication of appositional lymph-glands, but rather on the proof that actual generalization of the disease has taken place. When this has occurred such flesh must be unconditionally shut off from sale and consumption.

It is also an erroneous conclusion to assume that approaching emaciation is conclusive evidence of the extension of the tuberculous processes to the flesh, the latter only occurring when the disease has acquired the most extensive and destructive conditions in other and more important parts. It is well known that when the lungs, liver and intestines become the seat of tuberculous processes that marasimus soon sets in, without generalization, of necessity, having taken place. On the contrary, animals may have tuberculosis of the serosæ of a more or less extensive character for years and still continue in fair condition and be capable of being fattened, unless the disease extended to the important organs, and per metatosis, to other parts of the body. Therefore, there can be no generalization of the tuberculosis in a given animal when the virus has not gained access to the circulation, hence the flesh must be free.

The milk from tuberculous animals is to be considered as possessing an unquestionable infectious character. It must be the duty of the police to put a stop to the reckless usage of such milk to many babes. When the animals are known to have tuberculosis of the udder, the milk is to be at once destroyed.

Auscultation and prevention do not, even in extreme grades of tuberculosis, always give us trustworthy evidences of its nature.

Tubercular conglomerations of considerable size upon the pleura may escape recognition on account of the marked degree of resonans possessed by the thorax of cattle. The peculiar friction, noises, crepitus of this disease can only be heard when the plural tubercles have undergone calcification and their surface becomes more or less rough in consequence. Nymphomania, sterility and abortion, without visible cause in connection with approaching marasmus, are often connected with this disease, and have symptomatic value. These anomalies are only present when the tubercle deposits are principally situated in the peritonal regions, especially in the vicinity of the sexual organs. When the uterus is complicated a muco-purulent discharge often issues from the vagina. The axilliary inguinal and other accessible lymph-glands are often to be found in a hypertrophied condition. Diarrhœa of a chronic type is sometimes present. In young steers orchitis sometimes appears, apparently without cause. When the udder is affected we can frequently feel its lymphatics and nodules and cords, as well as indurated conditions of the glands. These, with chronic cough, nymphomania, etc., are sufficient to confirm the diagnosis.

Koch's methods of tuition for the diagnosis of tubercle bacilli have not as yet been accurately applied to the milk and discharge from tuberculous cows, hence their value as a means to diagnosis is yet to be decided. Intra ocular inoculations with the milk of suspected cows have not yet been, but should be, resorted to as an aid to diagnosis.

The so-called Milk Cures should be subjected to the most exact veterinary inspection, as well as milk farms supplying the cities, especially those places having few cows, which are kept in the suburbs and supply the poor in their immediate vicinity. The poor are generally the sufferers from dangerous and inferior articles of food.

It will be seen that veterinary police inspection, in order to gather statistics at markets, make practical observations over the transmissability and infectiousness of the disease in herds, also, in connection with the M.D., to watch results in families, to control the dairies, etc., is an absolute necessity in every country, and that every government which does not organize such a system is most emphatically not one for the people in any sense of the word.

### THE MANNER OF INFECTION IN TUBERCULOSIS.

Tuberculosis can be either acquired or hereditary; it can even be transmitted to the ovulum of the female by the generative act, or the primarily healthy embryo may become infected from the mother.

Although *intra*-uterine infection has been generally denied or doubted for human tuberculosis, it has been proven to occur, by undoubted observations, in tuberculosis in cattle and swine. It has been frequently observed in young animals of five or six weeks old; especially has this been the case in unborn or aborted fæti.

Chauveau, Konig, Eberhardt, and many others, have all given examples of intra-uterine tuberculosis in animals. The Bavarian veterinary reports are especially rich in such examples in both cattle and hogs.

### EXTRA-UTERINE INFECTION.

It is possible that the born animal can be infected.

We must hold to the fact that tuberculosis can be acquired through the digestive tract, from the consumption of the milk or flesh from infected animals.

It has been sufficiently proved that calves can be infected through the milk of tuberculous mothers. That the milk from cows, in which the udder is the seat of tubercular infiltration, contains a specific virus. These things have been proved by experiment and microscopical examination.

Gerlach declares that the infection of calves in this way is next to heredity, the most important etiological moment. Zippelius reports a fatal case of diarrhœa, in a calf with large tuberculous ulcerations in the intestines and tuberculosis of the viceral peritoneum ; the mother of the calf was slaughtered on account of the disease, which she had in a very severe form. Aufrech found the liver of a thirty-seven-days-old rabbit to be the seat of an extensive miliary tuberculosis, the mother of which was inoculated with virulent material a day after it gave birth to young. Others have reported similar results.

Such milk has also caused tuberculosis in swine fed upon it, and is especially prevalent in North German swine, where tuberculosis is also most frequently met with in cows. Observations have been resorted to where whole families of swine, held at dairies and cheese factories, have gradually perished from this cause. Frequently the disease seems to have appeared in the cattle and swine of the same form. As we have fully stated previously, the flesh of tuberculous cattle can produce the disease in other animals. In this consideration swine come chiefly into this catagory. Such flesh has been too frequently looked upon as a cheap variety of food for swine. If the sputum from such cattle has or can infect neighboring cows, or if the food becomes polluted with it, is still an open question.

Infection of healthy animals can take place through the inspired air through cohabitation, *i. e.*, through living with diseased animals.

This assertion has been so fully proved by the experiments of various authors, as to leave little doubt.

The assumption that the cohabitation of healthy and tuberculous cattle can cause the infection of the former, is becoming more and more confirmed since practical observation has been drawn to the subject.*

When such is the case in a herd the disease can only be eradicated by unconditionally slaughtering all the animals, and the thorough cleansing and disinfection of the stables before new ones are introduced.

The manner, or media, by which infection occurs in such cases, has not been settled.

### EXTRA-UTERINE INFECTION BY COITUS.

This is not doubted for human beings, and its possibility is to be assumed for animals, as uro-genital tuberculosis is by no means an uncommon occurrence in cows.

The predisposition to tuberculosis in individuals of certain descent is a factum of great etiological importance, and must not be neglected in the consideration of this question. As in man so in cattle we can assume the existence of local weaknesses, or an abnormal condition of certain tissues which especially favor the penetration and action of tuberculous virus. Such abnormal conditions can be either inherited or acquired, and be either rare or individual peculiarities.

Favoring such conditions have been suspected:

(*a.*) Too much feeding with swill foods and brewery refuse.

(*b.*) Continued confinement in poor, illy-ventilated, hot and moist stables, in which the animals are too-closely packed, and not allowed exercise.

Zippelius calls attention to the fact that tuberculosis is most frequently met with in cattle kept in deep valleys, and in such stables as described above.

---

* See original for cases, also the Translator's Book on Animal Diseases and Public Health for examples.

(*c.*) Forcing the cattle both in milk and calf production must necessarily produce debility and a tendency to this and other complications.

To the proper stamping out of this disease, the following regulations recommend themselves:

(*a.*) All tuberculous or animals with tubercular or tendencies to pulmonary diseases, must be unconditionally excluded from breeding.

(*b.*) All animals diseased with tuberculosis, must be unconditionally separated from healthy ones, and immediately slaughtered. Suspected ones should be treated in the same manner.

(*c.*) Stables in which such animals have been kept, must be thoroughly cleansed and disinfected.

(*d.*) All momenta tending to produce a predisposition to disease, must be carefully avoided, and great care given to ventilation, diet, exercise, exposure, etc.

